# Nurses’ priority-setting for older nursing home residents during COVID-19

**DOI:** 10.1177/09697330241226597

**Published:** 2024-02-05

**Authors:** My Eklund Saksberg, Therése Bielsten, Suzanne Cahill, Tiny Jaarsma, Ann-Charlotte Nedlund, Lars Sandman, Pier Jaarsma

**Affiliations:** 4566 Linköping University; 145651 Jönköping University; 8809 Trinity College Dublin; 4566 Linköping University; 4566 Linköping University; 4566 Linköping University; 4566 Linköping University

**Keywords:** COVID-19, Critical incident technique, Nursing, Nursing homes, Nursing priorities

## Abstract

**Background:**

Ethical principles behind prioritization in healthcare are continuously relevant. However, applying ethical principles during times of increased need, such as during the COVID-19 pandemic, is challenging. Also, little is known about nursing home nurses’ prioritizations in their work to achieve well-being and health for nursing home residents.

**Aim:**

The aim of this study was to explore nursing home nurses’ priority-setting for older nursing home residents in Sweden during the COVID-19 pandemic.

**Research design, participants, and research context:**

We conducted a qualitative interview study. Data were collected through in-depth interviews (retrospective self-reports) between February and May 2021 with 21 nursing home nurses. To help respondents to recall their memories, we used the critical incident technique (CIT). We analyzed data within the theoretical framework and the methodological orientation of content analysis.

**Ethical considerations:**

Written and verbal consent was obtained before the interviews, and information was given to participants informing them that participation was entirely voluntary. The Swedish Ethical Review Agency gave an advisory opinion stating that there were no ethical objections to the research project (Dnr. 2020-05649).

**Findings:**

We identified an overarching theme—nursing home nurses struggling on multiple fronts, “just do it”—and seven categories: striving for survival and caring about a dignified death; responding sensitively to relatives’ expectations; ranking the urgency of needed care; responding to input from different actors; combating the spread of infection in unconventional ways; taking the lead and doing what is required; and following the ideals of person-centered nursing.

**Conclusions:**

Nurses’ priority-setting for older nursing homes residents during the COVID-19 pandemic meant strain and struggle. In some cases, nurses had taken responsibility for priorities falling outside their statutory powers. Different demands and interests affected nurses’ priorities. Nursing home nurses need organizational and managerial support to prioritize.

## Introduction

Ethical principles of prioritization in healthcare are continuously relevant because the need for care often exceeds the resources available. Care needs may increase, such as during the COVID-19 pandemic, which brought healthcare rationing to the forefront of discussions on ethics.^1^ However, applying ethical principles during times of rationing is challenging.^2^

## Background

In Sweden, there are three statutory ethical principles for prioritizing healthcare: *the human dignity principle*; *the needs-solidarity principle*; and *the cost-effectiveness principle*.^3^ The application of these principles is based on directing resources to those with high care needs, based on measures that provide benefits to patients at a reasonable cost.^4^ Furthermore, the three statutory principles are applicable at various levels of the system: from overall priorities to the day-to-day clinical work of nurses as well as other healthcare professions.^5^

Nursing homes in Sweden, under the charge of municipalities, are characterized by home-like environments with social content and an emphasis on well-being.^6^ Although the medical aspect of the work is toned down compared to hospital care, the legal requirements for patient safety and quality are the same as those in hospitals.^7^ Nurses’ task is overall responsibility for nursing in this environment.^8,9^ The work is regarded as complex, as many of the residents suffer from multimorbidity.^10–12^ Assistant nurses, and healthcare assistants without formal education, make up most care team members, and their work consist mainly of basic nursing. The team also includes occupational therapists and physiotherapists, although in smaller proportions. Physicians—employed not by municipalities but by the region—make regular rounds.^6,13^

During the pandemic, conditions in nursing homes changed and circumstances became more demanding. In Sweden, hospital care provided by regional authorities was rationed because existing resources were not sufficient for all COVID-19 infected patients. The rationing also applied to residents of nursing homes.^14^ During this time, there was no overall joint support for priority-setting in nursing homes in Sweden or for nursing home nurses. Hence, circumstances may have caused nurses to prioritize differently during the COVID-19 pandemic than they may have during periods when resources were more constant and predictable and where situations were less challenging. For instance, various forms of interventions, such as preventing the spread of infection, may have led to different priorities and possibly resulted in the displacement of certain types of care.^15^

Although nurses prioritize as part of their daily work, knowledge of nurses’ experiences of priority-setting has been limited,^16^ and existing studies on nurses’ priority-setting mainly concern hospital care.^17^ There is a knowledge gap when it comes to nurses’ experience of priority-setting in nursing homes.^18^

Since we lack knowledge about nurses’ experiences of priority-setting among residents of nursing homes during times of rationing, there is reason to explore and describe this. Furthermore, knowledge from this study can contribute to the development of methods to support nurses, but also municipal healthcare providers, responsible politicians, and managers, to manage priorities in different situations to ensure high-quality safe care and fair distribution of resources. Therefore, we explored nursing home nurses’ priority-setting for older nursing home residents in Sweden during the COVID-19 pandemic.

## Methodological aspects

### Research design

We conducted a qualitative interview study where we sought to provide a deeper understanding of nurses’ actions, contextual factors, and the consequences of said actions.^19^ Data was collected through in-depth interviews, namely, retrospective self-reports. To help respondents recall their experiences, we used the critical incident technique (CIT).^20^ CIT helps individuals recall experiences of specific incidents at a level of detail where respondents can describe a time when a behavior, action, or occurrence impacted a specified outcome, positively or negatively.^21–23^

This study focused on the critical incident experiences narrated by nurses working in nursing homes for older people at the time of the COVID-19 pandemic. We have used the interpretation inspired by Butterfield et al., ^24^ that these critical incidents contain information about what happened at the beginning of the incident and what led up to the situation, and a thorough and detailed description of the situation itself and what happened afterward. Furthermore, specific real-life narratives were sought on events critical to the health and well-being of older people living in nursing homes in Sweden during the COVID-19 pandemic.

To avoid unnecessary confusion about the meaning of an adjective like “critical,” and to avoid the negative connotations of “incident” in healthcare contexts (e.g., patient falls), the researchers avoided the use of “critical” or any other adjective, such as “significant” or “meaningful,” and used “event” instead of “incident” in the interview questions.^25^

### Data collection and participants

Nurses were purposefully selected for participation in in-depth interviews through contact with managers of nurses working in nursing homes. Managers received verbal and written information about the study as well as informational letters, which included documentation regarding consent to participate in the study, to give to prospective respondents. All contacted organizations expressed interest in participating. Contact with the nurses was established and appointments for interviews were made.

Between February and May 2021, 21 registered nurses working in the care of older people, including one specialist nurse, from six different municipalities in southeast Sweden, participated in this study (Table 1). Participating nurses represented different ages and levels of experience. Some of the nurses were responsible for the care of older residents, while others were responsible for residents living in special units for persons with dementia.

**Table 1.** Characteristics of the sample (*n* = 21).

Gender
	Female	19
	Male	2
Age
	21–30	5
	31–40	4
	41–50	6
	51–60	4
	>60	2
Position
	Registered nurse	21
Years of nursing experience
	0–9	6
	10–19	8
	20–29	3
	30–39	2
	40+	2
Responsibility
	Older residents	6
	Residents with dementia	2
	Older residents and residents with dementia	13

To provide consistency in the interviews, an interview guide with semi-structured open-ended questions was used (Table 2). The interview guide, created by the authors, was based on questions formulated in a way that could help the respondent recall specific events in accordance with CIT principles.^22–25^ A pilot interview was conducted, and the questions in the interview guide were retained.

**Table 2.** Interview guide.

1. Can you tell me about an event (concerning a resident) where in retrospect you think that the outcomes would have been better if the situation was handled differently?
2. Can you tell me about an event (concerning a resident) where in retrospect you think that the outcomes were the best possible and where you believe the situation was handled the best way possible?
3. Can you identify and describe an event (concerning a resident) during the COVID-19 pandemic where you prioritized differently compared to pre-COVID-19?
4. Can you identify and describe an event (concerning a resident) during the COVID-19 pandemic where you did not prioritize differently than before COVID-19?
5. Can you describe an event (concerning a resident) during the COVID-19 pandemic in which it was difficult to prioritize?
6. Can you describe an event (concerning a resident) during the C OVID-19 pandemic in which it was easy to prioritize?
Follow-up questions (for each of the six questions):
1. What led up to the event?
2. What were your experiences of the event itself?
3. What was the outcome of the event?
4. What factors contributed to your prioritization?

The interviewer (MES) followed the guide to collect experiences of positive and negative events from each respondent to provide a comprehensive picture of experiences of prioritizations among older residents in nursing homes. The interviewer had the opportunity to change the order of the questions depending on the narrative and the topics introduced by the respondent, and the interviewer was also able to ask follow-up questions (Table 2).

Data were collected through one-to-one interviews with the presence of one interviewer (MES) and one participant. Fourteen interviews were conducted via video meetings, five were conducted face-to-face, and two were held on the phone. Participants were asked to be available for additional information in case any was needed. Interviews were digitally recorded and then transcribed verbatim. Respondents were informed that upon request, they would be provided with the interview transcripts.

### Data analysis

**Figure 1.**
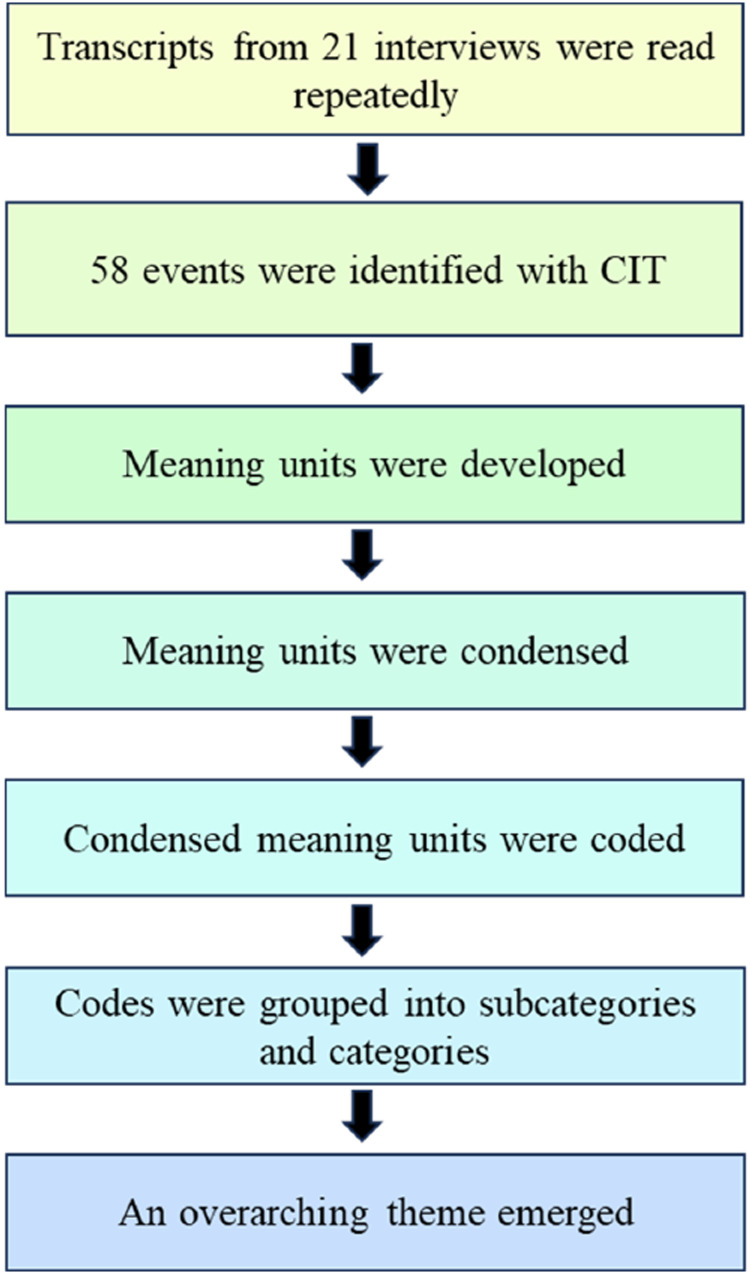
Analytical procedure.

We analyzed data within the theoretical framework and methodological orientation of content analysis, particularly conventional content analysis^26^ to systematically organize data into a structured format with the support of COREQ-guidelines.^27^ Transcripts were read repeatedly, and ambiguities were followed up by interceptions with recorded material. With the support of CIT, incidents were identified in the form of well-defined events,^25^ where nurses prioritized their work in relation to an older resident of the nursing home. A total of 58 events were identified. These parts—well-delineated events—of the textual material became the basis for analyzing data. Meaning units were developed corresponding to the research question and the purpose of the study. Subsequently, meaning units were condensed and encoded. Codes were then grouped into subcategories and categories which gave the manifest content. Through interpretation and analysis, an overarching, latent theme emerged. The procedure was repeated back and forth and iteratively until a satisfactory and trustworthy result was established. Two of the authors (MES and JP) performed the analysis through a predetermined procedure where each author followed the steps of the content analysis and where reconciliations were made together with the co-author after each step of the analysis (Figure 1).

### Ethical considerations

Written and verbal consent was obtained before the interviews, and information was given to participants informing them that participation was entirely voluntary. Given that the interviews could touch on emotionally difficult situations, measures were taken to protect respondents’ safety and security by allowing respondents to choose the location of the interview. To avoid breaking confidentiality, revealing information was removed. The Swedish Ethical Review Agency gave an advisory opinion stating that there were no ethical objections to the research project (Dnr. 2020-05649).

## Results

We identified an overarching theme—nursing home nurses struggling on multiple fronts, “just do it”—and seven categories: 1) striving for survival and caring about a dignified death; 2) responding sensitively to relatives’ expectations; 3) ranking the urgency of needed care; 4) responding to input from different actors; 5) combating the spread of infection in unconventional ways; 6) taking the lead and doing what is required; and 7) following the ideals of person-centered nursing.

### Theme: Nursing home nurses struggling on multiple fronts, “just do it”

Various forms of increased demands came to change the environment within which nurses set priorities: spread of infection; more extensive care needs of residents; lack of resources in the form of material; readiness and knowledge; unstable workforce; remote contact with worried and demanding relatives; remote consultations with physicians; and sometimes support from managers and sometimes not. Nurses talked about working under exceptional conditions and spoke about struggling and doing “the best one could,” and about prioritizing as “just do it.”

### Striving for survival and caring about a dignified death

The nursing home was described as an end station, where residents were expected to die in due course as part of normal life. But death became more pronounced during the pandemic as many residents died from COVID-19. The nurses talked about the importance of ensuring that the dying received palliative care: *“those patients who are assessed as being at the end of life and in need of palliative care, those patients have priority for me”* (Nurse 11). They also told that other tasks were put aside in favor of palliative care: *“well, when you realized that a resident wouldn’t survive, it was incredibly easy to put paperwork and other things aside”* (Nurse 15).

Paying attention to alleviating bothersome symptoms was a way to achieve well-being and dignity for dying residents. It was primarily arranged through medical interventions with focus on providing relief from pain, respiratory distress, nausea, and anxiety. But it emphasized also nursing such as arranging human presence with the dying resident. The nurses recounted incidents where they had succeeded, as well as hindering factors. There was an extensive effort to achieve the objectives of the palliative care as envisaged by the nurses, and sometimes their prioritizations were nearly impossible to implement: *“I must do as much as possible, sometimes I must turn myself inside out to make good for the resident”* (Nurse 11).

There were residents who fought for their lives and managed to survive, as well as events when nurses’ determination and efforts to save residents, despite rationing of hospital care, led to securing hospital care to residents who had a need for such resources: *“there was a resident who had difficulty breathing and I had to insist that we send her in to the hospital just to get oxygen. It was possible, but everyone questioned it. I think even the ambulance staff questioned it, but she survived, and she is fine today, too”* (Nurse 19).

### Responding sensitively to relatives’ expectations

The restrictions meant a ban on visits but that did not hinder communication with relatives. Nurses called relatives, and vice versa; the amount of phone calls was extensive. These conversations concerned keeping relatives informed and updated about the residents’ health: *“you want to keep the relatives up to date, and I would like to inform [them] about things that are happening”* (Nurse 4). It could give a sense of success, or a feeling of having executed the job well, when the relatives expressed satisfaction with the efforts undertaken. Nurses talked about that they had sensitivity for taking advantage of relatives’ wishes.

It happened that relatives became more demanding and assertive because they had clear ideas about what care their relative should receive. There were statements about the influence and power that relatives could have on the care provided. A particular resident’s care could be given special priority when relatives were insistent: *“I had incredible amount of contact with a family. They called all the time and questioned and wondered about the care. So, I really had to think about how to help the specific resident in the best way”* (Nurse 15).

### Ranking the urgency of needed care

Common priorities for achieving the well-being of a resident were medical assessments and decisions pertaining to the urgency of care measures. This form of prioritization was considered relatively manageable. Instantaneous events where a clear order of priority-setting was stated: *“in priority- setting one thinks breathing, bleeding and shock, and then underlying causes or diseases” (Nurse 19)*.

Occasionally, there were trade-offs between various priorities. Instances arose when the nurse was summoned for different cases simultaneously, necessitating an immediate decision on which individual’s care needs should take precedence*: “after all, if a resident has breathing problems, they need to be prioritized and cared for before a person who has fallen, unless the person who has fallen is bleeding from the head” (Nurse 2)*.

Conducting medical assessments for various conditions and prioritizing levels of care needs was straightforward, particularly in the case of prioritizing fall injuries: *“a lady fell and broke her hip, she couldn’t walk or stand, her leg was rotated and shortened. She was in great pain. It was easy to know what care she needed. It was a priority to send her off to the hospital” (Nurse 12)*.

### Responding to input from different actors

How nurses prioritize also stems from what information they are given. Some of the information comes through the daily dialogue with assistant nurses or healthcare assistants. Changes in the residents’ health were conveyed by them to the nurse, which could lead to new priorities.

It also happened that nurses’ autonomous professional practice became restricted by orders from the management. An example was an overall management decision that forced nurses (together with the physicians) to establish medical care plans for each resident in case the resident was infected with COVID-19. These plans, aimed at providing “good enough” care in the nursing home and reducing hospital missions. Because of perceived time pressure, these plans were drawn up without obtaining consent from the resident: *“it didn’t feel entirely good because I knew the backgrounds [of the plans] were set up without any consent”* (Nurse 16).

Questions about mandates arose. It happened that nurses, despite being competent in healthcare decisions, were not supported by management perceived to be lacking healthcare competence: *“I can prescribe extra personnel, relating to the need for human presence [for a dying, older resident], but management does not back it up because of the costs”* (Nurse 8).

### Combating the spread of infection in unconventional ways

Prioritizing the prevention of infection spread was essential, but protecting residents from being infected by COVID-19 became challenging. The extraordinary situation, with absence of preparedness and lack of resources, led to unusual priorities and solutions, for example, providing care and safeguarding the well-being of the resident, even if it meant the risk of becoming infected yourself: *“we lacked protective equipment, but I showed and told the other nurse to do this: turn your face away when the resident coughs”* (Nurse 1).

Nurses had to confront an unknown and deadly adversary, a virus about which there was significant apprehension. There was uncertainty about how the infection spread, and various directives with information about how care should be conducted were sent by superiors. The extensive information flows were burdensome*: “[…] We read everything in the beginning until we understood that we would drown in information”* (Nurse 1).

It was complicated to prevent the spread of infection among residents with dementia. Decisions made by the management could result in the adoption of controversial methods. In addition to some memos with information, decisions on priorities also came in the form of direct orders from superiors: *“all these guidelines, and the municipality chief nurse and physician who said: ‘This is how it should be.’ You just had to deal with it, but at the same time it was difficult ethically, having to sedate a resident”* (Nurse 16). Another way to reduce the spread of infection was to isolate other residents instead of the one who was infected: *“a person with dementia could not be isolated, he was difficult to isolate, and then we had to isolate the other residents quickly and as best we could to protect them, based on their approval”* (Nurse 3).

### Taking the lead and doing what is required

Priorities were made in circumstances characterized by solitary decisions. When work piled up during the pandemic, the nurses talked about “just do it”; take the lead without reflecting upon things too much: *“in the situation when things happen one simply acts. Since I (a nurse) had more experience than the staff, I took responsibility because the problem must be solved”* (Nurse 1).

The absence of physicians caused feelings of loneliness and of carrying a large burden of responsibility with little or no support. Due to the circumstances, nurses were forced to make decisions and prioritizations they usually are not responsible for making: *“[…] you stood there very alone. […] You really wanted to have the physician here to give a second opinion as well […] I prescribed all medications and judged that the resident should be cared for palliatively”* (Nurse 3). There was also uncertainty linked to medical assessments and additional concerns about the outcome of prioritizations and actions: *“it would have felt good if the physician had been involved […] I want the assessment to be proper”* (Nurse 3).

Beyond their typical duties, nurses also undertook basic nursing care tasks, ordinarily carried out by healthcare assistants or assistant nurses: for example, when the pandemic complicated the work and led to challenges, such as the uncertainty surrounding the spread of infection. In such cases, nurses did not hesitate to take the lead: *“…staff was very scared, terrified. Some were so scared that they cried. They really didn’t dare to go into the room of this lady [who had COVID-19 symptoms] …and we, the nurses, had to take care of this lady then, of course”* (Nurse 1).

The collaboration with management functions varied, which affected prioritization. Situations arose where nurses identified the need for actions, but these were not sanctioned by their superiors. However, other times there was a lack of management support and the priorities had to be implemented based on the nurse’s leadership and assessments of what was required.

### Following ideals of person-centered nursing

Accounts are given of various incidents where care was sensitively tailored to the preferences of the resident. This could also involve consulting different professionals, where teamwork took shape and where there was also cooperation with relatives.

Examples of responsiveness to the unique situation of certain residents became central to some of the prioritization. Assessments had been conducted, and a person-centered approach was applied: *“I took the time to listen to the resident, sat down, and allowed it to take time. I inquired about how she experienced the situation so that I could then assist her [...] in the best way possible, both in terms of nursing and medically”* (Nurse 14).

There were accounts of how residents, each with specific conditions, were treated with heightened sensitivity to foster a sense of safety and well-being, which came to play a role in some of the nurses’ priority-setting: *“there was a woman who had bad short-term memory and she often came out rolling from the room, even though she was not allowed to. And I mean such a person, you also must prioritize and speak to her about the situation, try to make her understand the problem” (Nurse 5)*.

Nurses recounted experiences where they carefully prioritized the care of certain residents regardless of the current situation (the ongoing pandemic). Feelings for certain individuals could also influence the work. With some residents, they had established longer and deeper relationships, which evoked their sympathies: *“I had a very close relationship with this man […] He was such a lovely person, we talked and laughed together daily”* (Nurse 9).

## Discussion

We obtained rich data and in-depth understanding in the present study about how nursing home nurses set priorities for the health and well-being of older nursing home residents in Sweden during the COVID-19 pandemic.

Our overarching theme—nursing home nurses struggling on multiple fronts, “just do it”—denotes the persistent work done by nurses during the pandemic. Nurses expressed that they had to face new challenges and increased workloads, which is in line with other qualitative studies,^28–30^ although there are also examples of the opposite, that is, that during the pandemic there has been a better work environment and less stress than otherwise.^31^ However, other studies highlight that stressful situations have seemed to be part of nursing home nurses’ work even under ordinary circumstances.^32–34^

To manage the amount of work, nurses create strategies, and to avoid the risk for creating additional work, nurses deprioritize some aspects of care, like neglecting visiting residents and by avoiding contact with relatives.^34^ Neglecting the care of older residential is a phenomenon that has received increasing attention.^35^ Our results differ. We found that nurses talked about the persistent struggle to keep up with all the often-instantaneously demanding work pouring over them. Only administration was mentioned as something they deliberately avoided. The same strategy has been described previously.^32,36^

Assistant nurses’ observations of residents’ health are of great importance. However, their priorities can differ from those of nurses, for example, they often identify with the residents.^37^ Personal sympathies may have an impact on which residents’ care is given a higher degree of urgency. In our study, some nurses have extra concerns about certain residents. The question is also nurses can identify themselves, as well as assistant nurses (Ibid) with certain residents and whether this in turn has consequences for how nurses prioritize.

We found that nurses took responsibility for the work of other professionals. Nurses in the present study transgressed their formal competence and authority when performing certain tasks reserved for physicians. In these cases, it was a matter of prioritizing tasks to secure medical care that nurses judged residents needed. But there were also top-down decisions about priorities where nurses were forced to act against their better judgment. Nurses felt bad when they were compelled to perform actions that violated their ethical code^38^ and beliefs about residents’ rights. Front-line nurses, during the pandemic, handled similar moral dilemmas with moral courage and compassionate care to safeguard the best interests of patients , despite the fact that they were at risk of moral distress.^39^

In healthcare, every situation involves a number of choices and prioritizations, and trade-offs arise.^40^ Prioritization can lead to postponing or even rationing of non-basic or more complex nursing care tasks regarding the needs of other residents. It also gives rise to implicit priorities when some measures are chosen over others, which in turn are linked to omitted care and patient safety risks.^41^

Particularly important for nurses was striving to provide good palliative care, to reduce or alleviate bothersome symptoms,^42^ and to ensure residents their right to a dignified death, which is consistent with frameworks for palliative care,^43–45^ and the nurses’ ethical code.^38^ It is conceivable that routines regarding palliative care played a role in the nurses’ priorities. Statements in our findings can also stand for thinking about a practice or desired practice based on current policies.^46^

Nurses set priorities between different individuals based on their instantaneous care needs. Ranking the urgency of different medical conditions was something nurses seemed to be confident about. Similar patterns have been described previously.^47^

We found, in line with nursing ethics,^38^ that nurses prioritized creating good relationships with relatives, although it was time-consuming. There is a reciprocity; relatives usually desire to be informed about their elderly relative’s well-being.^48–50^ This desire has also been described in palliative care.^51,52^ But we found a risk that the relative who shouts the loudest will be able to access more healthcare resources.

There were managerial decisions about priorities that the nurse had to follow, even though they ran counter to the nurse’s ethical beliefs and autonomous profession. Priorities are not just an issue for the individual employee but for the entire system.^41^ Practitioners’ knowledge should be considered at all levels when setting priorities because there is specific knowledge that has been formed through proximity to decision-making that directly affects the patient and the ethical foundations of healthcare.^53^ Lack of support but also ignorance regarding ethical priorities—that is, aggravating contextual conditions—have been discussed on nurses’ and physicians’ priority-setting in nursing homes.^54^ There are studies that point to managers’ lack of knowledge on the ethical principles found in healthcare legislation, that ethical principles have a low status in decision-making, and that ethics are separated from management.^55^

Monteverde^56^ describe similar focus like ours, especially in the early phase of the pandemic, on preventing the spread of infection in nursing homes. A contemporary study found ethical dilemmas like ours for nurses during the COVID-19 pandemic, such as having the sole responsibility for patient care or the use of unconventional methods to ensure certain well-being and health of residents.^57^

## Limitations

There is always a risk of diminished trustworthiness when narrating from memories that depend on the human mind. CIT is a method that minimizes the risk of warping,^21^ but there remains a risk that respondents talked of things that are not fully ascertainable as an objective, measurable truth. On the other hand, these are characteristics of qualitative methods that do not seek to find answers through quantification and generalization.^26^

We should be aware of the risk of bias based on the ongoing large media coverage during the pandemic. There have been experiences of feeling criticized or being questioned as a front-line worker in nursing homes.^58^ To what extent it affected respondents is difficult to know. It should be considered that society-changing events, such as pandemics, may also have affected researchers.^59^

Interviews may be regarded as reciprocal, co-creative processes wherein knowledge is exchanged in both directions, influenced by the interviewer’s involvement. Respondents’ reflections are integral in promoting sense-making and shaping new strategies for future practices. These perspectives, offered by Nardon et al.,^60^ align with Schön’s reflective model.^61^ This model emphasizes that reflections on actions, as exemplified by respondents in this study, can foster learning and lead to the refinement of work practices. However, a potential drawback of the critical incident technique (CIT) is that its title, along with its focus on extraordinary events, may cause apprehension among respondents. It has been observed that nurses interviewed using CIT tend to avoid discussing events related to their skills and actions, preferring instead to discuss incidents outside their scope of competence.^23^ In our data collection, the interview guide (Table 2) has proved helpful, particularly the follow-up questions designed to identify incidents that specifically relate to the respondents’ own priorities.

Finally, we would like to consider that in our results, some categories overlap and are thus not pure, which should be the aim of performance reporting in content analysis.^62^ Furthermore, our categories bear names that answer the question “how,” which is not customary in content analysis.^63^ Nevertheless, the names adequately reflect the manifest content of the categories.

## Conclusions

Nursing home nurses in Sweden have experienced strain and struggled to prioritize the well-being of older residents during the COVID-19 pandemic. They faced demands from actors with different interests that can be based on both “non-principles” and statutory ethical principles for prioritization. Furthermore, nurses narrated how they often handle prioritizations alone by “just doing it”, with increased responsibility incorporating the tasks of other professions.

Nursing home nurses need to have both theoretical knowledge of laws governing priorities, method support, and judgment, as well as the skills needed to apply priorities in a legally secure manner. It is also important that caregivers and managers support nurses’ application and implementation of the statutory ethical principles of priorities in practice. Finally, we recommend continued research on nurses’ and other professionals’ priority-setting in nursing homes.
